# Prenatal phenotypes and pregnancy outcomes of fetuses with recurrent 16p13.11 microduplications

**DOI:** 10.1097/MD.0000000000049059

**Published:** 2026-06-05

**Authors:** Tangfei Xu, Fagui Yue, Yao Ge, Ruizhi Liu

**Affiliations:** a Center for Reproductive Medicine, Center for Prenatal Diagnosis, First Hospital, Jilin University, Changchun, China; b Jilin Engineering Research Center for Reproductive Medicine and Genetics, Jilin University, Changchun, China.

**Keywords:** 16p13.11 microduplications, chromosome microarray analysis, genotype–phenotype correlation, prenatal diagnosis

## Abstract

16p13.11 microduplication is a frequently observed chromosomal abnormality in newborns. However, prenatal reports for this chromosome microscopic imbalance are rare in clinical practice. This study aimed to provide a systematic summary of prenatal phenotypes for this genomic disorder. Between April 2019 and May 2023, 10 pregnant women underwent amniocentesis due to fetal ultrasound abnormalities, advanced maternal age, or other prenatal diagnostic indications. After obtaining informed consent, chromosomal microarray analysis and G-banding were performed. Karyotype results were normal for all fetuses except in 2 cases (P2 and P5). Chromosomal microarray analysis detected 0.79 to 1.639 Mb duplications of 16p13.11 (chr16: 14806186–16444739, hg38) in all 10 cases, involving 4 morbid genes (*NDE1, MYH11, ABCC1*, and *ABCC6*) in common. Eight women (P1–P8) continued their pregnancies and delivered healthy infants at term, while the parents of cases 9 and 10 terminated their pregnancies. All neonates exhibited good health states without obvious abnormalities observed. The prenatal phenotypes of 16p13.11 duplications were primarily associated with abnormal soft markers, including increased nuchal translucency, ventriculomegaly, and enhanced intestinal echo. However, further evidence is needed to explore prenatal genotype–phenotype correlations. For prenatally diagnosed cases carrying 16p13.11 microduplications, long-term follow-up should be carried out to acquire their postnatal health conditions and growth details.

## 1. Introduction

Chromosomal microscopic imbalances, such as microduplications, are usually associated with intellectual disability, growth retardation, autism spectrum disorder (ASD), and other related disorders.^[1]^ As is well known, chromosome microarray analysis (CMA) has been recognized as a first-tier approach and gold standard for detecting disease-causing copy number variation (CNV) in prenatal and postnataldiagnosis due to its superior sensitivity and high efficiency.^[2]^ With the widespread application of CMA in clinical practice, a series of CNVs with clinical significance have been detected, such as 22q11.21, 17q12, 16p13.11, 1q21.1, 15q13.3, and 10q21.1.^[3]^

As an unbalanced structural variant, 16p13.11 duplications could be subdivided into 3 intervals (I, II, and III), each of which is flanked by sequences rich in low copy repeats, also called segmental duplications.^[4,5]^ For 16p13.11 microduplication, it was initially regarded as a rare benign variant. However, increasing evidence delineated that this chromosomal microscopic imbalance might be associated with a series of clinical features, including attention-deficit hyperactivity disorder, intellectual disability, developmental delay, ASD, schizophrenia, epilepsy, congenital heart defects, and skeletal malformations.^[5–7]^ In addition, some studies reported mildly affected and phenotypically normal members sharing the same 16p13.11 duplicated locus in the same family, which would probably be due to the incomplete penetrance and variable expressivity for this chromosome disorder.^[6,7]^

To date, most studies on the CNV spectrum in the 16p13.11 region were diagnosed postnatally. Prenatal reports involving 16p13.11 microduplications were limited. To better understand the 16p13.11 microduplications in the prenatal setting, we present the clinical and molecular findings of 10 prenatal cases with 16p13.11 microduplications using CMA, and provide a systematic summary of prenatal phenotypes for such genomic disorders.

## 2. Materials and methods

### 2.1. Subjects

This retrospective study was performed from April 2019 to May 2023 and enrolled 10 cases with 16p13.11 microduplications selected from 11,547 pregnant women. The main indications for prenatal diagnosis included noninvasive prenatal testing (NIPT) for aneuploidy, maternal serum screening results for aneuploidy, ultrasound anomalies (structural or nonstructural), parental chromosomal abnormalities, abnormal childbearing history, advanced maternal age, and voluntary request. All pregnant women accepted routine prenatal ultrasound examinations during the gestation period, and abnormal ultrasound findings were included in the indications for prenatal diagnosis. Detailed clinical information is summarized in Table 1. All parents were healthy, non-consanguineous, and free of congenital anomalies. All women denied gestational alcohol intake, as well as exposure to teratogens, radiation, and infectious diseases during pregnancy. This study protocol was approved by the Ethics Committee of the First Hospital of Jilin University (2021-706) and written informed consents were obtained from all couples for publication of this case report and accompanying images.

**Table 1:** Summary of the cytogenetic, chromosomal microarray analysis, and clinical findings of our cases with 16p13.11 duplication.

Case	Sex	Pregnancy history	Gestational age	Birth		Karyotype results	CMA results (arr[GRCh38])	Duplicated size (Mb)	Inheritance	Duplicated gene	Prenatal diagnosis indications/reason of study	Half a year postpartum follow-up
				Weight (kg)	Length (cm)							
P1	M	G2P1	39w	3.5	50	46,XN	16p13.11(15060499–16215189)x3	1.155	*de novo*	*MYH11*, *NDE1*	AMA	Live birth; no evident anomalies
P2	M	G1P0	37w3d	3.9	50	46,XN,21pss	16p13.11(15387890–16215189)x3	0.827	pat	*MYH11*, *NDE1*	NIPT indicated a high risk of chromosome 16	Live birth; no evident anomalies
P3	M	G3P1	37w2d	2.95	49	46,XN	16p13.11(14957887–16414266)x3	1.456	pat	*MYH11*, *NDE1*	AMA	Live birth; no evident anomalies
P4	M	G1P0	40w	3.75	52	46,XN	16p13.11(15405588–16195202)x3	0.79	pat	*MYH11*, *NDE1*	Increased NT	Live birth; no evident anomalies
P5	M	G1P0	37w2d	2.58	49	NA	16p13.11(15387891–16184275)x3	0.796	pat	*MYH11*, *NDE1*	Ultrasound findings inferred choroid plexus cyst and echogenic foci in the ventricle	Live birth; no evident anomalies
P6	M	G2P0	40w	3.25	50	46,XN	16p13.11(14964966–16215189)x3	1.25	NA	*MYH11*, *NDE1*	Increased NT	Live birth; no evident anomalies
P7	M	G1P0	39w4d	3.3	50	46,XN	16p13.11(14806186–16444739)x3	1.639	pat	*MYH11*, *NDE1*	Increased NT	Live birth; no evident anomalies
P8	M	G3P1	38w3d	3.5	52	46,XN	16p13.11(15282055–16215189)x3	0.933	mat	*MYH11*, *NDE1*	ART	Live birth; no evident anomalies
P9	NA	G3P1	TOP at 23w	NA	NA	46,XN	16p13.11(15387891–16189012)x3	0.801	NA	*MYH11*, *NDE1*	Increased NT	TOP
P10	NA	G1P0	TOP at 27w3d	NA	NA	46,XN	16p13.11(14835214–16195202)x3	1.36	*de novo*	*MYH11*, *NDE1*	AMA, ultrasound findings inferred a single umbilical artery in the fetus	TOP

Genomic parameters are from GRCh38/hg38.

AMA = advanced maternal age, ART = assisted reproductive technology, d = days, F = female, M = male, mat = maternally inherited, NA = not available, NIPT = noninvasive prenatal testing, NT = nuchal translucency, pat = paternally inherited, TOP = termination of pregnancy, w = weeks.

### 2.2. Cytogenetic analysis

G-banding karyotyping was conducted at a resolution of 300–400 bands, following the method described in our previous work.^[8]^ Metaphase spreads were prepared from both amniotic fluid cells and peripheral blood lymphocytes using conventional cytogenetic protocols. A minimum of 20 metaphases were examined per sample. All karyotypes were interpreted and reported in accordance with the International System for Human Cytogenetic Nomenclature 2020.

### 2.3. Chromosomal microarray analysis

After obtaining written informed consent from each pregnant woman, 10 mL of uncultured amniotic fluid was sampled via amniocentesis. Genomic DNA was extracted with the Qiagen Micro Kit (Qiagen) following the standard protocol. CMA was performed on the Affymetrix CytoScan 750K (Affymetrix, Santa Clara) array platform. The experimental procedure strictly adhered to the manufacturer’s guidelines and the previously described protocol.^[9]^ The process included genomic DNA extraction, digestion and ligation, polymerase chain reaction amplification and product purification, quantification and fragmentation, labeling, array hybridization, washing, and scanning. Then the Chromosome Analysis Suite V4.2 software (Affymetrix) was used for analyzing the CEL files. Genome-wide screening thresholds were set at ≥100 kb for copy number gains and losses. The identified CNVs were interpreted by integrating evidence from peer-reviewed literature and the following public databases:

1. Database of Genomic Variants (http://dgv.tcag.ca/dgv/app/home),
2. DECIPHER (https://www.deciphergenomics.org/),
3. Online Mendelian Inheritance in Man (OMIM, http://www.ncbi.nlm.nih.gov/omim),
4. UCSC (https://genome.ucsc.edu/),
5. ClinGen (https://www.clinicalgenome.org/), and
6. PubMed (https://pubmed.ncbi.nlm.nih.gov/).

Genomic positions refer to the Human Genome Assembly December 2013 (GRCh38/hg38).

### 2.4. Statistical analysis

The SPSS 26.0 software (IBM Corp.) was used to conduct a comparative analysis of the prenatal phenotypes of 16p13.11 microduplication cases between both our study and the published literature, using either the χ^2^ test or the Fisher exact test. During the analysis, a *P*-value < .05 was considered statistically significant.

## 3. Results

**Figure 1.**
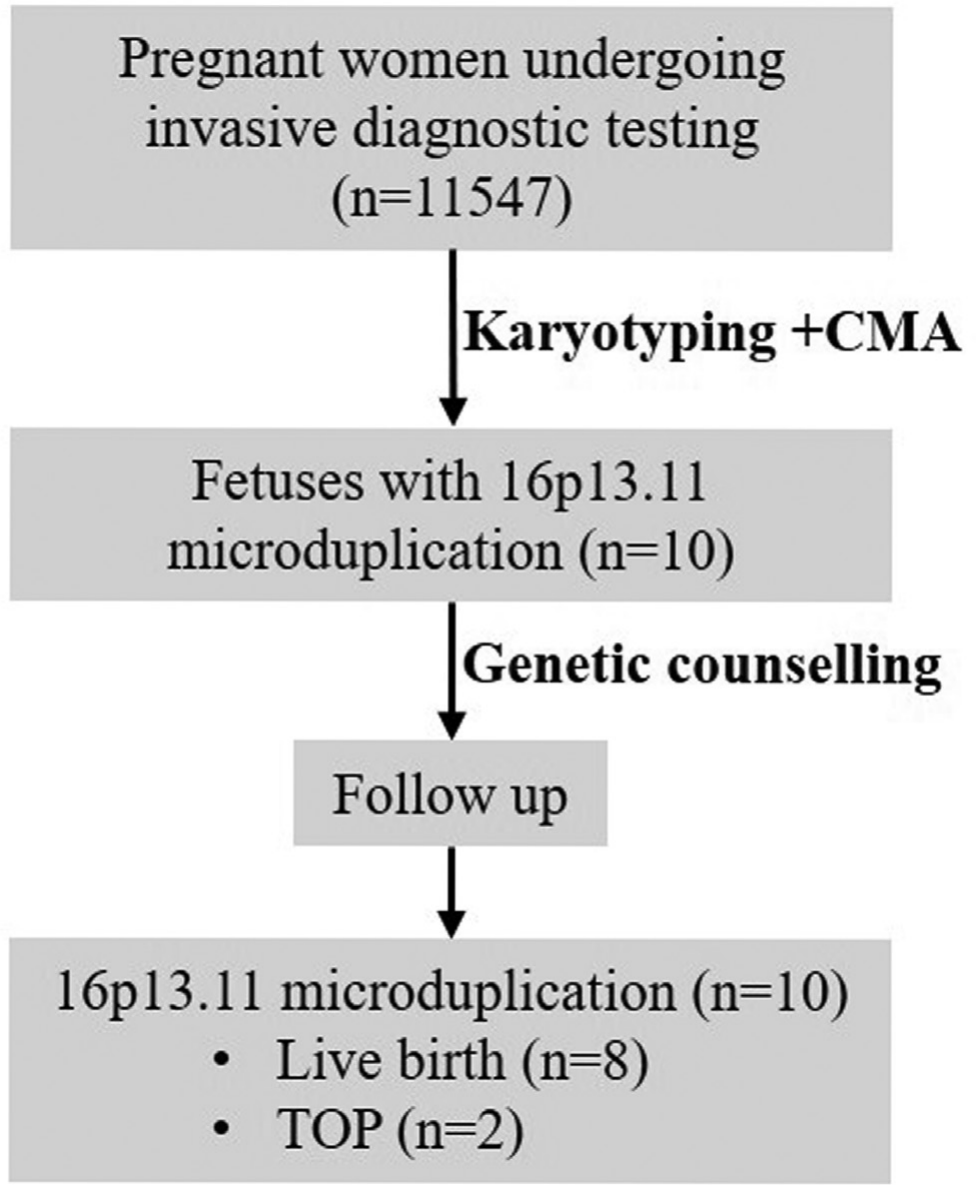
The flowchart of the study. CMA = chromosome microarray analysis, TOP = termination of pregnancy.

**Figure 2.**
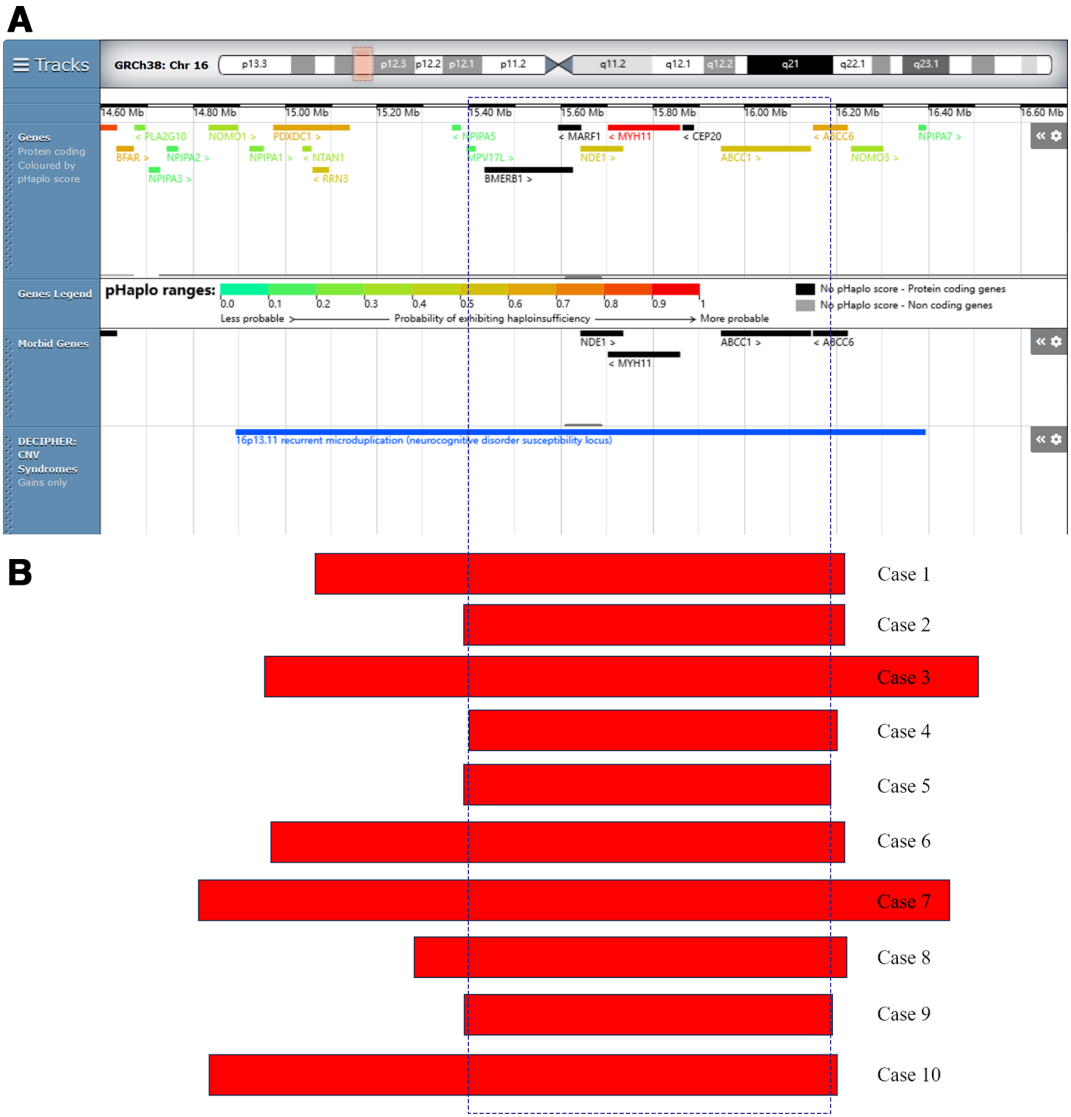
Scale representation of the duplicated region in the short arm of chromosome 16p13.11 (https://www.deciphergenomics.org/): (A) location of genes in the region and (B) duplicated fragments in the present cases (red) in the region. Genomic parameters are from GRCh38/hg38. CNV = copy number variation.

Of 11,547 pregnant women opting for prenatal invasive testing, 10 fetuses were identified with 16p13.11 microduplications, with a total detection rate of 0.10% (10/11,547) in the prenatal setting. Detailed clinical data and follow-up outcomes are shown in Figure 1. The duplication sizes ranged from 0.79 to 1.639 Mb (Fig. 2). All cases shared similar 16p13.11 microduplications involving 4 morbid genes. The distributions of indications for prenatal diagnosis were as follows: increased nuchal translucency (NT; 4/10), advanced maternal age (3/10), abnormal NIPT result (1/10), choroid plexus cyst (1/10), echogenic foci in the ventricle (1/10), a single umbilical artery in the fetus (1/10), and assisted reproductive technology (1/10). Conventional karyotyping showed normal results in all fetuses except for case P2 (chromosomal polymorphism) and case P5 (cell culture failure). Detailed cytogenetic, CMA, and clinical findings are presented in Table 1. Except for P6 and P9, the other couples opted for CMA to identify the origins of the duplications. Paternal inheritance was confirmed in 5 fetuses (P2–P5, P7), maternal inheritance in 1 fetus (P8), and *de novo* occurrence in 2 fetuses (P1, P10). The parents of P9 and P10 chose to terminate their pregnancies based on genetic counseling. Postnatal follow-up was conducted for all live-born cases, focusing on congenital defects, developmental delay, physical growth, craniofacial dysmorphisms, and skeletal abnormalities. To date, none of these infants have shown obvious anomalies; however, long-term follow-up is still necessary.

### 3.1. Literature review

To further elucidate the prenatal genotype–phenotype correlations of 16p13.11 microduplications, we conducted a literature review on previously reported prenatal cases harboring similar 16p13.11 duplications (Table 2).^[10–19]^ The duplications across these cases ranged from 0.6 to 3.27 Mb, all encompassing the 16p13.11 region. Among these cases, 14 patients were paternally inherited, 15 were maternally inherited, 5 were *de novo*, and 36 were not available. Pregnancy outcomes: 49/70 live births, 7/70 termination of pregnancy, 2/70 ongoing, and 12/70 unavailable. Among these duplications, 26/70 cases presented fetal ultrasound abnormalities, 22/70 cases presented abnormal maternal serum screening results, 7/70 cases presented advanced maternal age, 6/70 cases presented abnormal NIPT results, and 9/70 were unavailable. Among the fetal ultrasound abnormalities, the recurrent prenatal phenotypes of duplication include ventriculomegaly (5/70), enhanced intestinal echo (4/70), increased NT (3/70), fetal growth restriction (3/70), ventricular septal defect (2/70), persistent left superior vena cava (2/70), and mild tricuspid regurgitation (2/70). The rate of increased NT detection in our study was significantly higher than that in the literature review (40.0% vs 4.3%, *P* < .05).

**Table 2:** Clinical data of fetuses presenting 16p13.11 microduplication in the published literature.

No.	Sex/age	Duplicated region	Duplicated size	Inheritance	Prenatal diagnosis			References
					CMA results (arr[GRCh38])	Indications for 16p13.11 microduplication	Follow-up	
1	NA	16p13.11	0.92	NA	16p13.11(15231215–16178546)x3	FUA: urorectal septal malformation sequence	TOP	Cai et al^[10]^
2	F/3 yr	16p13.11	0.78	NA	16p13.11(15416655–16215189)x3	FUA: mild tricuspid regurgitation	Well survivor	
3	M/4 yr	16p13.11	1.25	NA	16p13.11(14964963–16215189)x3	FUA: enhanced intestinal echo	Well survivor	
4	F/4 yr	16p13.11	1.25	pat	16p13.11(14964963–16215189)x3	FUA: ventriculomegaly	Well survivor	
5	M/3 yr	16p13.11	1.6	NA	16p13.11(14806185–16444739)x3	Balanced translocation of chromosomes in the father of the fetus	Well survivor	
6	NA	16p13.11	1.6	NA	16p13.11(14806185–16414266)x3	FUA: enhanced intestinal echo	TOP	
7	F/2 yr	16p13.11	1.6	NA	16p13.11(14799118–16444739)x3	FUA: right renal duplication	Well survivor	
8	F/1.5 yr	16p13.11	1.65	NA	16p13.11(14827007–16444739)x3	Balanced translocation of chromosomes in pregnant women	Well survivor	
9	F/1.5 yr	16p13.11	1.12	mat	16p13.11(15060499–16189012)x3	FUA: lung cystadenomatous lesions	Postnatal lung cystadenoma surgery without other abnormalities	
10	M/4 mo	16p13.11	1.3	NA	16p13.11(14835213–16178546)x3	MSS: high risk for Down’s screening	Well survivor	
11	M/1.3 yr	16p13.11	0.77	pat	16p13.11(15387890–16178546)x3	FUA: increased NT	Well survivor	
12	F/1.2 yr	16p13.11p12.3	2.92	NA	16p13.11p12.3(15231215–18148856)x3	MSS: high risk for Down’s screening	Well survivor	
13	M/1.1 yr	16p13.11	1.48	NA	16p13.11(14964963–16444739)x3	FUA: enhanced intestinal echo	Well survivor	
14	M/8 mo	16p13.11	0.6	mat	16p13.11(15603678–16215189)x3	FUA: ventriculomegaly	Well survivor	
15	M/6 mo	16p13.11	1.2	NA	16p13.11(15060499–16215189)x3	AMA	Well survivor	
16	NA	16p13.11p12.3	3.23	NA	16p13.11p12.3(15310619–18620668)x3	FUA: increased NT (NT = 2.9 mm)	NA	Coello-Cahuao et al^[11]^
17	M/3 yr	16p13.11	3.27	pat	16p13.11(15310625–18658374)x3	NA	Neonatal jaundice	Kang et al^[12]^
18	M/4 yr	16p13.11	1.26	mat	16p13.11(14954924–16217184)x3	NA	Well survivor	
19	F/4 yr	16p13.11	1.26	pat	16p13.11(14954924–16217184)x3	NA	Well survivor	
20	F/3 yr	16p13.11	1.08	pat	16p13.11(14954924–16033991)x3	NA	Well survivor	
21	M/NA	16p13.11	1.16	*de novo*	16p13.11(15055129–16217184)x3	NA	NA	
22	M/4 yr	16p13.11	1.23	pat	16p13.11(14874998–16100721)x3	NA	Well survivor	
23	M/NA	16p13.11	1.05	pat	16p13.11(15055099–16100721)x3	NA	NA	
24	M/NA	16p13.11	1.83	pat	16p13.11(14816371–16648326)x3	FUA: cleft lip	TOP	
25	M/3 yr	16p13.11	1.83	mat	16p13.11(14816371–16648326)x3	NA	Well survivor	
26	NA	16p13.12p12.3	1.38	NA	16p13.12p12.3(15060499–16435824)x3	FUA: brain, cardiovascular system	NA	Kang et al^[13]^
27	NA	16p13.11	1.63	NA	16p13.11(14799119–16433802)x3	MSS: high risk of 21 trisomy	NA	
28	NA	16p13.11	1.62	mat	16p13.11(14827008–16444739)x3	MSS: high risk of 21 trisomy	NA	
29	NA	16p13.11	0.8	pat	16p13.11(15387891–16189012)x3	FUA: digestive system	NA	
30	NA	16p13.11	0.85	NA	16p13.11(15387891–16234030)x3	MSS: high risk of 21 trisomy	NA	
31	NA	16p13.12p12.3	1.06	NA	16p13.12p12.3(15387890–16444739)x3	FUA: cardiovascular system	NA	
32	NA	16p13.11	3.06	mat	16p13.11(15084745–18148856)x3	MSS: high risk of 21 trisomy	NA	
33	NA	16p13.11	1.3	NA	16p13.11(14799118–16184276)x3	MSS: high risk of 21 trisomy	TOP	Chen et al^[14]^
34	NA/2 yr	16p13.11	0.89	pat	16p13.11(14964963–16184276)x3	AMA	Well survivor	
35	NA/2 yr	16p13.11	0.85	pat	16p13.11(15387890–16234030)x3	FUA: fetal double top diameter, head circumference less than gestational age	Well survivor	
36	NA/9 mo	16p13.11	1.4	mat	16p13.11(14960453–16414266)x3	AMA	Well survivor	
						Abnormal childbearing history		
37	NA	16p13.11	1.6	NA	16p13.11(14799119–16444739)x3	FUA: the fetal femur diameter was <2 standard deviations of gestational age, the humerus was less than the lower limit of normal, and the tricuspid valve regurgitation was mild (FGR, tricuspid mild reflux)	TOP	
38	NA	16p13.11p12.3	2.97	NA	16p13.11p12.3(15100125–18078611)x3	AMA	Well survivor	Zhang et al^[15]^
39	NA	16p13.11p12.3	1.95	NA	16p13.11p12.3(14806185–16764475)x3	MSS: high risk for Down’s screening (1/245)	Well survivor	
40	NA	16p13.11p12.3	2.81	mat	16p13.11p12.3(15265282–18078611)x3	MSS: high risk for Down’s screening (1/173)	Well survivor	
41	NA	16p13.11	1.64	NA	16p13.11(14799118–16444739)x3	MSS: high risk for Down’s screening (1/54)	Well survivor	
42	NA	16p13.11	0.79	*de novo*	16p13.11(15387890–16184276)x3	MSS: high risk for Down’s screening (1/32)	Well survivor	
43	NA	16p13.11	1.6	NA	16p13.11(14827007–16435824)x3	MSS: high risk for 18 trisomy	Well survivor	
44	NA	16p13.11	2.67	NA	16p13.11p12.3(15387890–18057820)x3	NIPT: a 3.6-Mb duplication was present at 16p13.11p12.3	Well survivor	
45	NA	16p13.11	1.37	NA	16p13.11(14806185–16178546)x3	MSS: high risk for Down’s screening (1/153)	Well survivor	
46	NA	16p13.11	1.05	NA	16p13.11(15376894–16431526)x3	AMA	Well survivor	
47	NA	16p13.11	1.64	NA	16p13.11(14799118–16444739)x3	NIPT: high risk for 16 trisomy	Well survivor	
48	NA	16p13.11	1.02	mat	16p13.11(15387890–16414266)x3	MSS: high risk for Down’s screening (1/204)	ongoing pregnancy	
49	NA	16p13.11	1.38	NA	16p13.11(14835213–16215189)x3	FUA: increased NT	ongoing pregnancy	
50	NA	16p13.11	1.21	NA	16p13.11(14964963–16184276)x3	FUA: left superior vena cava, dilated coronary sinus, ventricular septal defect, abnormal course of venous catheter	TOP	
51	NA	16p13.11	0.79	NA	16p13.11(15405588–16195202)x3	NIPT: duplication of chromosome 16	Well survivor	Luo et al^[16]^
52	NA	16p13.11p12.3	2.8	*de novo*	16p13.11p12.3(15244295–18078611)x3	FUA: fetal broadening the left ventricle, FGR	TOP	
53	NA	16p13.11	1.2	mat	16p13.11(14964963–16178546)x3	MSS: high risk of 18 and 21 trisomy	Well survivor	
54	NA	16p13.11	1.4	mat	16p13.11(15045272–16444739)x3	MSS: high risk for Down’s screening	Well survivor	
55	NA	16p13.11	1.2	pat	16p13.11(14964964–16178546)x3	NIPT: microdeletion of chromosome 7	Well survivor	
56	NA	16p13.11	0.79	NA	16p13.11(15387891–16178546)x3	MSS: high risk for Down’s screening	Well survivor	
57	NA	16p13.11p12.3	2.7	mat	16p13.11p12.3(15225421–18058629)x3	NIPT: microdeletion of chromosome 16	The right ear hearing loss	
58	NA	16p13.11p12.3	2.8	mat	16p13.11p12.3(15244296–18071186)x3	NIPT: abnormality of chromosome 16	Well survivor	
59	NA	16p13.11	1.7	NA	16p13.11(14964964–16215189)x3	FUA: anterior mitral valve crack	Well survivor	
60	NA	16p13.11	1.2	pat	16p13.11(14964964–16184276)x3	FUA: persistent left superior vena cava	Well survivor	
						AMA		
						MSS: high risk for Down’s screening		
61	NA	16p13.11	1.4	*de novo*	16p13.11(14799119–16234030)x3	MSS: high risk for Down’s screening	Well survivor	
						FUA: fetal single umbilical artery		
						Abnormal childbearing history		
62	NA	16p13.11	1.3	pat	16p13.11(14960453–16215189)x3	FUA: fetal ventricular septal defect	Ventricular septal defect	
63	NA	16p13.11	1.3	*de novo*	16p13.11(14964964–16215189)x3	MSS: high risk for Down’s screening	Well survivor	
64	NA	16p13.11	1.1	NA	16p13.11(15282055–16414266)x3	Both parents beta thalassemia	Well survivor	
65	NA	16p13.11p12.3	1.5	mat	16p13.11p12.3(16636518–18148856)x3	MSS: high risk for Down’s screening	Well survivor	
66	NA	16p13.11p12.3	2	mat	16p13.11p12.3(14799119–16764475)x3	AMA	Well survivor	
67	NA	16p13.11	1.16	NA	16p13.11(15046143–16206143)x3	MSS: abnormal results	Slightly delayed growth and development	Huang et al^[17]^
68	NA	16p13.11p12.3	1.96	NA	16p13.11(14806185–16764475)x3	FUA: broadening of the cerebral ventricles, corpus callosum dysplasia, expansion of the third ventricle, brain dysplasia, FGR	NA	Li et al^[18]^
69	NA	16p13.11p12.3	1.97	NA	16p13.11(14799118–16764475)x3	FUA: broadening of the cerebral ventricles, enhanced ventricular wall, and intestinal echo	NA	
70	F/NA	16p13.11	0.82	NA	16p13.11(15386143–16206143)x3	NA	Umbilical hernia	Deng et al^[19]^

Genomic parameters are from GRCh38/hg38.

AMA = advanced maternal age, d = days, F = female, FGR = fetal growth restriction, FUA = fetal ultrasound abnormalities, M = male, mat = maternally inherited, MSS = maternal serum screening, NA = not available, NIPT = noninvasive prenatal testing, NT = nuchal translucency, pat = paternally inherited, TOP = termination of pregnancy, w = weeks.

## 4. Discussion

We described 3 rare prenatal cases with pure 16p13.11 microduplications, ranging from 0.79 to 1.639 Mb. Five fetuses (P2–5, P7) were identified as paternal inheritance, 1 (P8) was identified as maternal inheritance, and 2 (P1, P10) showed *de novo* duplications. To our knowledge, reports of prenatally detected 16p13.11 microduplications are rare in clinical practice. All cases involved in our study presented diverse ultrasound findings, ranging from normal to abnormal.

Chromosomal duplications are implicated in a broad spectrum of human genetic disorders.^[20]^ 16p13.11 microduplications are commonly associated with multiple clinical phenotypes, mainly neurocognitive and behavioral disorders, including intellectual disability, ASD, schizophrenia, epilepsy, and attention-deficit/hyperactivity disorder. In addition to the neurological manifestations, 16p13.11 microduplications have also been associated with congenital heart defects, familial thoracic aortic aneurysms, and dissections. Most clinical phenotypes were documented in adults and children. Given the incomplete penetrance of this chromosome disorder, prenatal diagnosis and consultation for 16p13.11 microduplications are challenging.

**Figure 3.**
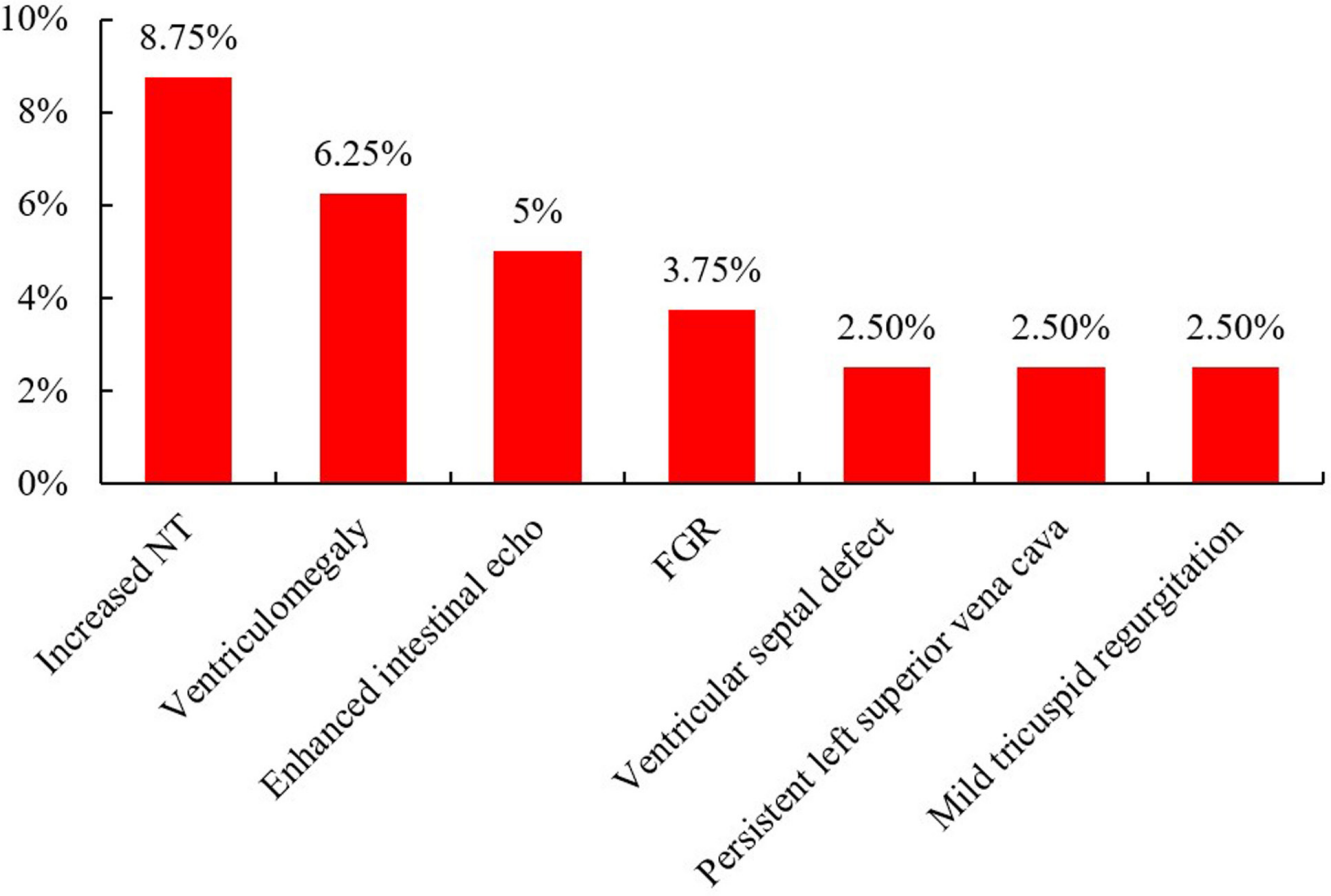
The frequencies of 16p13.11 prenatal ultrasound phenotypes. FGR = fetal growth restriction, NT = nuchal translucency.

According to the literature review and the results of our study, the summarized frequencies for recurrent ultrasound findings associated with 16p13.11 duplications are as follows: increased NT (7/80), ventriculomegaly (5/80), enhanced intestinal echo (4/80), fetal growth restriction (3/80), ventricular septal defect (2/80), persistent left superior vena cava (2/80), and mild tricuspid regurgitation (2/80) (Fig. 3). Based upon the findings mentioned above, we assumed that 16p13.11 duplications were closely associated with increased NT, ventriculomegaly, and enhanced intestinal echo.

According to the DECIPHER database, 4 morbid genes are located in the 18p11.32 region detected in our cases (Table 3), which are correlated with diverse diseases. As is known, the haploinsufficiency of genes would result in genetic disorders. In order to predict the potential genes associated with prognostic phenotypes, we delineated their functions and implications in different processes.

**Table 3:** Overlapping genes in the 16p13.11 region in our case.

Gene	OMIM	Description	Disease
*NDE1*	609449	NudE neurodevelopment protein 1	Lissencephaly 4; microhydranencephaly
*MYH11*	160745	Myosin heavy chain 11	Aortic aneurysm, familial thoracic 4; megacystis-microcolon-intestinal hypoperistalsis syndrome 2; visceral myopathy 2
*ABCC1*	158343	ATP-binding cassette subfamily C member 1	Deafness, autosomal dominant 77
*ABCC6*	603234	ATP-binding cassette subfamily C member 6	Arterial calcification, generalized, of infancy, 2; pseudoxanthoma elasticum; pseudoxanthoma elasticum, forme fruste

ATP = adenosine triphosphate, OMIM = Online Mendelian Inheritance in Man.

The *NDE1* gene (OMIM: 609449), containing 9 exons, interacts with *Lis1* to promote activation of dynein motility.^[21]^ It encodes a scaffold protein essential for brain development.^[22–24]^ Biallelic *NDE1* loss of function causes microcephaly with profound mental retardation. However, *NDE1* missense mutations and CNVs are associated with multiple neuropsychiatric disorders.^[25]^ Myosin heavy chain 11, encoded by the *MYH11* gene (OMIM: 160745), is a protein that participates in muscle contraction through the hydrolysis of adenosine triphosphate.^[26]^ Pathogenic variants in *MYH11* can reduce aortic structural integrity and contractility, leading to aortic dissection.^[27]^ In addition, the *MYH11* variant is also associated with megacystis-microcolon-hypoperistalsis syndrome and visceral myopathy.^[28]^ *ABCC1* (OMIM: 158343) contains 31 exons and is a member of the ABC transporter family. It encodes multidrug resistance protein 1, which transports a structurally diverse range of endogenous substances, as well as xenobiotics and their metabolites. Li et al reported that *ABCC1* variants are associated with hereditary deafness.^[29]^ *ABCC6* (OMIM: 603234) belongs to the multidrug resistance-associated protein subfamily of adenosine triphosphate-binding cassette transmembrane transporters. Mutations in the *ABCC6* gene cause pseudoxanthoma elasticum, which is a heritable connective tissue disorder characterized by calcification of elastic fibers in skin, arteries, and retina. According to the ClinGen database and literature review, there is still no supporting evidence of triplosensitivity for these genes (triplosensitivity score: 0) till now.

Considering the phenotypic diversity of 16p13.11 microduplications and the young age of our cases, it is unclear whether delayed clinical symptoms will emerge in the future. Therefore, long-term follow-up involving their postnatal health and growth details is necessary. Moreover, the samples included in the study were from a single center, and the cohort size was relatively small. Hence, multicenter collaborations should be adopted to enlarge the sample size to establish a clearer correlation between 16p13.11 microduplications and prenatal phenotypes.

## 5. Conclusion

We identified 10 prenatally diagnosed cases with 16p13.11 microduplications using CMA. Fetuses with 16p13.11 microduplications could exhibit diverse ultrasound findings, ranging from normal to abnormal. Abnormal ultrasound soft markers are a common prenatal phenotype of 16p13.11 microduplications, mainly including increased NT, ventriculomegaly, and enhanced intestinal echo. For postnatal cases with 16p13.11 microduplications, long-term monitoring of physical and developmental growth from childhood to adulthood is clinically essential.

## Author contributions

**Conceptualization:** Tangfei Xu, Ruizhi Liu.

**Data curation:** Fagui Yue.

**Formal analysis:** Yao Ge.

**Supervision:** Ruizhi Liu.

**Writing—original draft:** Tangfei Xu.

**Writing—review & editing:** Fagui Yue.
